# STAT2-dependent restriction of Zika virus by human macrophages but not dendritic cells

**DOI:** 10.1080/22221751.2021.1929503

**Published:** 2021-06-08

**Authors:** Dong Yang, Hin Chu, Gang Lu, Huiping Shuai, Yixin Wang, Yuxin Hou, Xi Zhang, Xiner Huang, Bingjie Hu, Yue Chai, Terrence Tsz-Tai Yuen, Xiaoyu Zhao, Andrew Chak-Yiu Lee, Ziwei Ye, Cun Li, Kenn Ka-Heng Chik, Anna Jinxia Zhang, Jie Zhou, Shuofeng Yuan, Jasper Fuk-Woo Chan

**Affiliations:** a State Key Laboratory of Emerging Infectious Diseases, Department of Microbiology, The University of Hong Kong, Pokfulam, People’s Republic of China; b Key Laboratory of Tropical Translational Medicine of Ministry of Education, Hainan Medical University, Haikou, People’s Republic of China; c Hainan-Medical University-The University of Hong Kong Joint Laboratory of Tropical Infectious Diseases, Hainan Medical University, Haikou, Hainan, People’s Republic of China, and the The University of Hong Kong, Pokfulam, People’s Republic of China; d Department of Pathogen Biology, Hainan Medical University, Haikou, Hainan, People’s Republic of China; e Carol Yu Centre for Infection, Li Ka Shing Faculty of Medicine, The University of Hong Kong, Pokfulam, People’s Republic of China; f Department of Microbiology, Queen Mary Hospital, Pokfulam, People’s Republic of China; g Department of Clinical Microbiology and Infection Control, The University of Hong Kong-Shenzhen Hospital, Shenzhen, People’s Republic of China

**Keywords:** Dendritic cells, flavivirus, macrophages, interferon signaling, STAT1, STAT2, Zika

## Abstract

Zika virus (ZIKV) is an emerging mosquito-borne flavivirus that poses significant threats to global public health. Macrophages and dendritic cells are both key sentinel cells in the host immune response and play critical roles in the pathogenesis of flavivirus infections. Recent studies showed that ZIKV could productively infect monocyte-derived dendritic cells (moDCs), but the role of macrophages in ZIKV infection remains incompletely understood. In this study, we first compared ZIKV infection in monocyte-derived macrophages (MDMs) and moDCs derived from the same donors. We demonstrated that while both MDMs and moDCs were susceptible to epidemic (Puerto Rico) and pre-epidemic (Uganda) strains of ZIKV, virus replication was largely restricted in MDMs but not in moDCs. ZIKV induced significant apoptosis in moDCs but not MDMs. The restricted virus replication in MDMs was not due to inefficient virus entry but was related to post-entry events in the viral replication cycle. In stark contrast with moDCs, ZIKV failed to inhibit STAT1 and STAT2 phosphorylation in MDMs. This resulted in the lack of efficient antagonism of the host type I interferon-mediated antiviral responses. Importantly, depletion of STAT2 but not STAT1 in MDMs significantly rescued the replication of ZIKV and the prototype flavivirus yellow fever virus. Overall, our findings revealed a differential interplay between macrophages and dendritic cells with ZIKV. While dendritic cells may be exploited by ZIKV to facilitate virus replication, macrophages restricted ZIKV infection.

## Introduction

Zika virus (ZIKV) is an emerging mosquito-borne flavivirus that has raised global concerns in recent years [1]. In addition to the transmission of ZIKV by *Aedes* mosquito, it can also be sexually and vertically transmitted from person to person. While most ZIKV-infected patients are asymptomatic or mildly symptomatic, some adult patients develop severe neurological complications such as Guillain–Barré syndrome, and fetuses may develop congenital microcephaly and anomalies [1,2]. Since 2015, ZIKV has rapidly spread to over 87 countries and territories and remains a potential risk of future epidemics as new lineages continue to emerge [3,4].

Macrophages and dendritic cells are key antigen-presenting cells that play pivotal roles in the pathogenesis of flavivirus infections. They act as mediators of innate and adaptive immunity. Depletion of macrophages in types I and II interferon (IFN) receptor-deficient mice results in a significantly higher level of systemic dengue virus (DENV) titer [5]. Mice lacking type I IFN receptor expression on CD11^+^ dendritic cells and LysM^+^ macrophages develop fatal DENV infection [6]. Dendritic cells prime B and T cell responses against flavivirus infections via upregulation of major histocompatibility complex (MHC) and co-stimulatory molecules such as CD80, CD83, and CD86, as well as production of cytokines and chemokines [7–9]. Moreover, macrophages and dendritic cells are direct targets of DENV, West Nile virus, and Japanese encephalitis virus (JEV) that support virus replication and dissemination as Trojan horses [10–15]. In mosquito-borne transmission, DENV infects macrophages, resident CD103^+^ classical dendritic cells (cDCs), and Ly6C^-^/CD11b^+^ cDCs, and recruits monocytes to further differentiate into inflammatory dendritic cells and macrophages [16,17]. Importantly, certain tissue-resident macrophages derived from monocytes have been illustrated to be susceptible to ZIKV infection [18]. For example, ZIKV is able to efficiently infect and replicate in primary placental macrophages (Hofbauer cells), resulting in dissemination across the placental barrier and vertical transmission [19,20]. In the dermis, blood monocytes differentiate into monocyte-derived macrophages (MDMs) or monocyte-derived tissue-resident macrophages [16,21]. Mosquito-borne flaviviruses may therefore be able to target these cells to facilitate virus dissemination.

IFN signaling plays essential roles in protecting the host from viral infection. Flaviviruses possess IFN antagonism strategies to suppress the host IFN response and facilitate virus replication. ZIKV infection impairs type I and III IFN signaling and suppresses the induction of downstream interferon-stimulated genes (ISGs) through virus-mediated STAT2 degradation [22-24]. Moreover, ZIKV antagonizes type I IFN signaling through blockade of STAT1 and STAT2 phosphorylation and productively infects human monocyte-derived dendritic cells (moDCs) [24-26]. Despite extensive studies on flavivirus antagonism, the role of IFN signaling upon ZIKV infection in human MDMs remains incompletely defined.

Extending on these preliminary findings, in the present study, we utilized human primary MDMs as a model to investigate the role of macrophages in ZIKV infection. Our results demonstrated that ZIKV replication in MDMs was largely restricted compared to that in moDCs. We further found that ZIKV infection in MDMs did not efficiently suppress type I IFN-mediated antiviral responses and failed to inhibit IFN-α-induced STAT1 and STAT2 phosphorylation. Importantly, depletion of STAT2 but not STAT1 in MDMs significantly rescued ZIKV and yellow fever virus (YFV) replication. Collectively, our results indicated that human MDMs restrict flavivirus replication through a STAT2-dependent mechanism. These differential interplays between MDMs and moDCs with ZIKV provided novel insights into our understanding on the virus-host interaction in ZIKV infection.

## Materials and methods

### Cells and viruses

VeroE6 was purchased from the American Type Culture Collection (ATCC) and maintained in Dulcecco’s Modified Eagle’s Medium (DMEM), supplemented with 10% fetal bovine serum (FBS), 100 units/mL of penicillin and 100μg/mL of streptomycin (5%CO_2_ at 37°C). ZIKV^PR^ (Puerto Rico strain PRVABC59) was obtained from a patient in the recent South American epidemic (kindly provided by Brandy Russell and Barbara Johnson, Centers for Disease Control and Prevention, USA) and ZIKV^U^ (ZIKVAF-976 Uganda strain) was isolated from a nonhuman primate in Uganda in 1947 (kindly provided by Tatjana Avšič Županc, University of Ljubljana, Slovenia, the European Virus Archive) [27,28]. Yellow fever virus 17D (YFV) was available in our laboratory and cultured as previously described [28].

### Preparation of human primary monocyte-derived macrophages (MDMs) and monocyte-derived dendritic cells (moDCs)

Healthy volunteer blood samples were collected from Hong Kong Red Cross Blood Transfusion Service according to a protocol approved by the Institutional Review Board of the University of Hong Kong. Human peripheral blood mononuclear cells (PBMCs) were isolated from the buffy coats as we previously described [29]. MDMs were differentiated from PBMCs by providing RPMI-1640 supplemented with 10% FBS, 100 units/mL of penicillin, 100μg/mL of streptomycin, 1% sodium pyruvate, 1% non-essential amino acids, 1U/mL GM-CSF (Cell Sciences, Canton, MA, USA) [30]. MoDCs were differentiated and maintained as the same condition as MDMs with IL-4 (R&D systems) [31].

### Virus infections

MDMs and moDCs were differentiated in complete RPMI-1640 with specific growth factors for 6–7 days before infection. For immunofluorescence microscopy experiment, MDMs and moDCs were uninfected or infected with ZIKV^PR^ or ZIKV^U^ [multiplicity of infection (MOI) = 1.0] for 2 h at 37°C. After 2 h incubation, the virus inoculum was washed off and the cells were maintained with fresh RPMI-1640. For flow cytometry analysis, MDMs and moDCs were uninfected or infected with ZIKV^PR^ or ZIKV^U^ (MOI=0.1 or MOI=1.0) for 2 h at 37°C. For viral replication kinetics evaluation, cells were infected with ZIKV^PR^, ZIKV^U^, or YFV (MOI=0.01 or 1.0) for 2 h at 37°C. For extended period replication kinetics and cell viability evaluation, the cells were infected with ZIKV^PR^ or ZIKV^U^ (MOI=0.1) for 2 h at 37°C. For IL-13 treatment experiment, after 24 h treatment with IL-13, the cells were infected with ZIKV^PR^ or ZIKV^U^ (MOI=0.01) for 2 h at 37°C. For virus entry capability experiment, the cells were incubated with ZIKV^PR^ at an MOI of 10.0 in an ice bath for 2 h, followed by incubation with 37°C for 30 min. After 30 min incubation, the virus inoculum was washed off and the cell lysates were collected for further detection. For ISGs evaluation experiment, the cells were uninfected or infected with ZIKV^PR^ (MOI=10.0) for 6 h, followed by mock treatment or treatment with IFN-α for 6 h. For Western blots experiment, the cells were infected with ZIKV^PR^ (MOI=1.0) for 1 h at 37°C. For small interfering RNA (siRNA) knockdown experiment, after transfection with siRNA, the cells were infected with ZIKV^PR^ or YFV (MOI=1.0) for 1 h at 37°C.

### Immunofluorescence staining and confocal microscopy

Immunostaining and confocal microscopy were performed as we previously described [32,33]. Briefly, MDMs and moDCs cultured in 24-well plate were digested by trypsin and re-seeded into chamber slide. After one day of culture in the RPMI-1640 with growth factors, the cells were uninfected or infected with ZIKV^PR^ or ZIKV^U^ (MOI=1.0) for 2 h at 37°C. At 48hpi, the infected MDMs and moDCs were washed with phosphate-buffered saline (PBS) and fixed in 4% paraformaldehyde. The cells were permeabilized for 10 min with 0.2% Triton X-100 and blocked with Dako blocking buffer for 30 min at room temperature. The cells were subsequently incubated with mouse 4G2 monoclonal antibody (MERCK) against envelope (E) protein of pan-flavivirus or mouse J2 monoclonal antibody (SCICONS) against double-stranded RNA (dsRNA) diluted with Dako antibody diluent overnight at 4°C, followed by incubation with goat anti-mouse Fluor 488 immunoglobulin G (IgG) (Abcam) secondary antibody diluted with Dako antibody diluent for 1 h. The nuclei of the cell were stained by DAPI nucleic acid stain (Thermo Fisher Scientific) for 10 min. The slides were imaged with confocal microscopy using a Carl Zeiss LSM 880 system (Zeiss, Oberkochen, Germany).

### Flow cytometry analysis

Flow cytometry was performed according to standard protocols as we previously described [34,35]. In brief, MDMs and moDCs were infected with ZIKV^PR^ or ZIKV^U^ (MOI=0.1 or MOI=1.0) for 2 h at 37°C. The infected cells were detached with EDTA at 24hpi and 48hpi and fixed with 4% paraformaldehyde. The cells were then permeabilized for 10 min with 0.2% Triton X-100 and blocked with PBS supplemented with 2% FBS. The cells were incubated with primary antibodies (4G2 or J2) for 1 h at 4°C, followed by incubation with secondary antibody (goat anti-mouse Fluor 488 IgG) for 45 min at 4°C. Multi-color flow cytometry acquisition was performed using the FACS Canto II Analyzer (BD Biosciences), and the data were analyzed by FlowJo software.

### RNA extraction and quantitative reverse transcription polymerase chain reaction (qRT-PCR)

Cellular RNA extraction, reverse transcription, and quantitative PCR were performed as we described previously [36]. For evaluation of viral genome copy in supernatants and cell lysates, real-time qRT-PCR was used to quantify ZIKV^PR^ or ZIKV^U^ genome copy with LightCycler 96 Real-Time PCR System (Roche) as we described previously [37,38]. For host genes analysis, the cells were lysed using the RLT buffer provided from RNA extraction kit (TAKARA). The extracted RNA was reverse transcribed with Transcriptor First Strand cDNA Synthesis Kit (Roche). The levels of cellular gene expression were normalized to GAPDH and presented as per GAPDH in gene expressions.

### Virus replication kinetics

MDMs, moDCs, and VeroE6 (2 × 10^5^ cells per well of 24-well plate) were infected with ZIKV^PR^, ZIKV^U^, or YFV [MOI=0.01 (2 × 10^3^ PFU) or MOI=1.0 (2 × 10^5^ PFU)] for 2 h at 37°C and washed with PBS three times, followed by incubation with fresh medium. The cell lysates and supernatants were harvested for determining the genome copy and live infectious virus by qRT-PCR and plaque assays. For extended period viral replication kinetics, the cells were infected with ZIKV^PR^ or ZIKV^U^ (MOI=0.1) for 2 h at 37°C. The supernatants were collected at the indicated days post infection for determining virus titer by plaque assays.

### Evaluation of cell viability and caspase-3/7 activity

Cell viability and Caspase-3/7 activity of mock- or virus-infected MDMs and moDCs were determined using CellTiterGlo assays (Promega, Madison, USA) and CaspaseGlo 3/7 assays (Promega, Madison, USA), respectively [39]. Briefly, the cells were infected with ZIKV^PR^ or ZIKV^U^ (MOI=0.1) for 2 h at 37°C. The cells were lysed together with culture supernatants at a 1:1 ratio with the CellTiterGlo reagent or CaspaseGlo 3/7 reagent at the indicated days post infection and placed on an orbital shaker for 10 min to induce cells lysis. The plates were read by measuring the luminescence signal with the VectorX3 multi-label plate reader (PerkinElmer, Waltham, MA, USA) as we previously described [40].

### IL-13 treatment of MDMs

MDMs were untreated or treated with IL-13 of 20 and 50 ng/mL for 24 h and the cell lysates were harvested for testing expression of ZIKV entry-related host factors by qRT-PCR. MDMs treated with IL-13 of 50 ng/mL and moDCs treated with the same amount of DMSO were infected with ZIKV^PR^ or ZIKV^U^ (MOI=0.01). The cell lysates of the infected cells were collected at 24hpi for determining the viral genome copy by qRT-PCR.

### Evaluation of viral entry capability

MDMs and moDCs were incubated with ZIKV^PR^ (MOI=10.0) in an ice bath for 2 h, followed by incubation at 37°C. After 30 min incubation, the virus inoculum was discarded and the cells were washed three times with PBS. The cell lysates were collected for further detection.

### Recombinant human IFN-α treatment

MDMs and moDCs were uninfected or infected with ZIKV^PR^ (MOI=10.0) for 6 h at 37°C. After 6 h, the cells were subsequently washed with PBS and were untreated or treated with 1000U/mL of recombinant human IFN-α (PBL Assay Science). After 6 h of treatment, the cell lysates were harvested for detecting expression of interferon-stimulated genes (ISGs) by qRT-PCR.

### Western blots and analysis

MDMs and moDCs were infected with ZIKV^PR^ (MOI=1.0) at 37°C. At 48hpi, the cells were untreated or treated with 1000U/mL of recombinant human IFN-α for 30 min and then lysed with RIPA buffer supplemented with Protease Inhibitor Cocktail and Phosphatase Inhibitor Cocktail II (Thermo Fisher Scientific). The expression levels of STAT1, STAT2, STAT1 phosphotyrosine residue 701 (pSTAT1), STAT2 phosphotyrosine residue 689 (pSTAT2), and β-actin were detected using anti-STAT1 (BD Transduction Laboratories), anti-STAT2 (SANTA CRUZ BIOTECHNOLOGY), anti-pSTAT1 (Cell Signaling Technology), anti-pSTAT2 (Millipore), and anti-β-actin (Sigma-Aldrich) primary antibodies. The intensity of the bands was calculated using Image J software to generate the histogram. For evaluation of knockdown efficiency of siRNA transfection, MDMs and moDCs were transfected with scrambled siRNA or siRNA targeting STAT1 or STAT2. The expression of STAT1, STAT2, and β-actin were detected by Western blots.

### Co-immunoprecipitation (co-IP) assays

The co-IP assays were performed using Pierce Crosslink Magnetic IP/co-IP kit according to manufacturer’s instruction (Thermo Fisher Scientific). Briefly, MDMs and moDCs were infected with ZIKV^PR^ at an MOI of 5.0. After 24hpi, the whole cell lysates were incubated with protein A/G magnetic beads pre-crosslinked with anti-ZIKV NS5 antibody (GeneTex) or anti-STAT2 antibody (SANTA CRUZ BIOTECHNOLOGY). After incubation, the supernatants and beads were magnetically separated. The elution buffer from the kit was added into the beads and magnetically separated. The supernatants were harvested for detecting the expression of ZIKV NS5, STAT2, and β-actin by Western blots as we described earlier.

### Transfection of siRNA

MDMs and moDCs were generated from two different healthy donors. The cells were transfected with siRNA targeting STAT1 or STAT2, as well scrambled siRNA as a negative control, followed by infection with ZIKV^PR^ at an MOI of 1.0 as we previously described [41]. At 2hpi and 24hpi, the cell lysates were harvested for determining viral genome copy by qRT-PCR and the data was presented as fold change in viral genome copy of siRNA targeting STAT1 or STAT2 transfected cells relative to scrambled siRNA transfected cells. The supernatants were harvested for determining live infectious virus titer by plaque assays.

### siRNA knockdown and Poly IC treatment

MDMs and moDCs were transfected with 70nM siRNA targeting STAT1 or STAT2 obtained from Dharmacon using RNAiMAX (Thermo Fisher Scientific), with scrambled siRNA as negative controls, followed by transfection with 5ug of Poly IC per well. After 12 h, the cell lysates were harvested for detecting host gene expressions by qRT-PCR, including IFIT1, MX1, ISG15, IRF1, and IP10.

### Statistical analysis

The data are presented as mean ± standard deviation. Student’s t-test and one-way ANOVA were used for statistical analysis. Differences were considered significant when *p* < 0.05. * indicated *p* < 0.05, ** indicated *p* < 0.01, *** indicated *p* < 0.001, and **** represented *p* < 0.0001.

## Results

### ZIKV replication is supported by moDCs but restricted in MDMs

**Figure 1.**
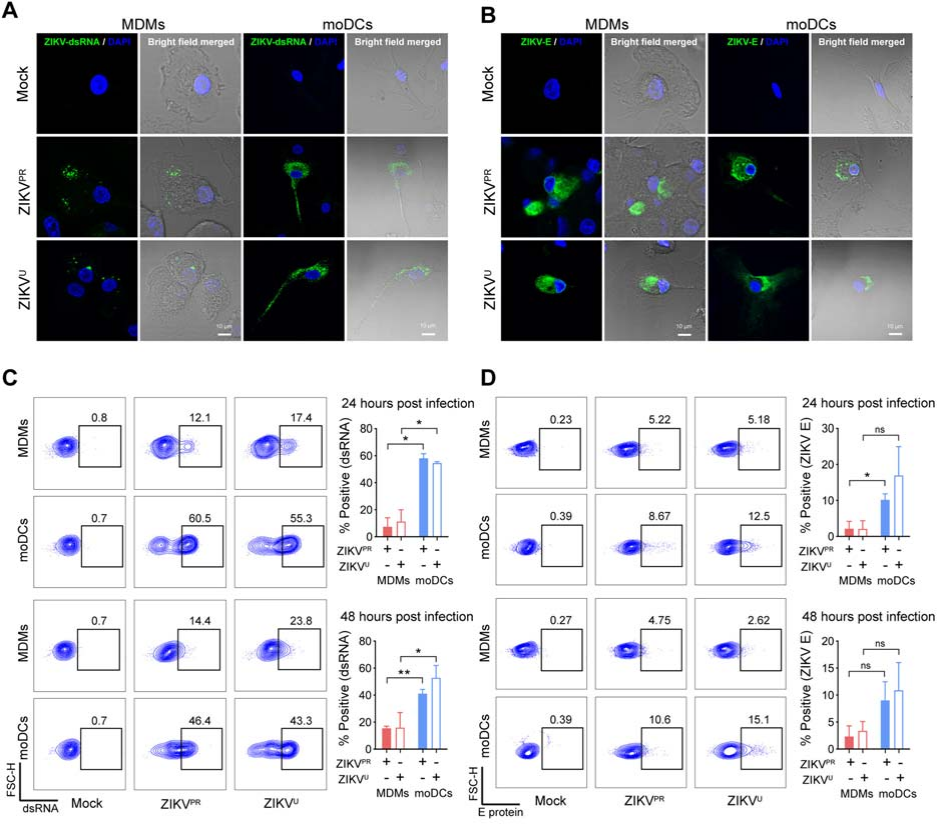
**MDMs are susceptible to ZIKV infection.** Human monocyte-derived macrophages (MDMs) and dendritic cells (moDCs) were uninfected or infected with ZIKV^PR^ or ZIKV^U^ at an MOI of 1.0. At 48 h post-infection (hpi), the cells were fixed with 4% paraformaldehyde and then permeabilized with 0.2% Triton X-100. **(A)** Viral double-stranded RNA (dsRNA) and **(B)** envelope (E) protein were identified with J2 and 4G2 antibodies, respectively, and imaged with confocal microscopy. In parallel, MDMs and moDCs were infected with ZIKV^PR^ or ZIKV^U^ at an MOI of 0.1. At 24hpi and 48hpi, ZIKV-infected and mock-infected cells were fixed and stained with J2 or 4G2, and subsequently applied to flow cytometry to determine expression levels of **(C)** viral dsRNA and **(D)** E protein. The dot-plot showed data of one representative donor. Statistical analyses in all panels were performed with Student’s t-test and the differences were considered significant when *p* < 0.05. *p < 0.05, **p < 0.01. ns, not significant. The histogram showed the mean values and standard deviations from three donors. Bars represented 10 μm.

In this study, we used human primary MDMs and moDCs from healthy donors as models of peripheral macrophages and dendritic cells, respectively. First, we tested the susceptibility of MDMs to ZIKV infection using ZIKV^PR^ or ZIKV^U^. moDCs from the same donors were included for comparison. Confocal microscopy revealed that viral double-stranded RNA (dsRNA) diffusely distributed as small puncta in the cytoplasm of ZIKV^PR^- or ZIKV^U^-infected MDMs (Figure 1A). The expression of ZIKV envelope (E) protein was detected to predominantly accumulate at the perinuclear regions in MDMs upon infection (Figure 1B). The distributions of viral dsRNA and E protein were similar in ZIKV^PR^-infected and ZIKV^U^-infected MDMs. The overall cellular distribution pattern of viral dsRNA and E protein in ZIKV-infected MDMs largely resembled that of ZIKV-infected moDCs (Figure 1A and 1B).

We further quantitatively examined ZIKV infection in MDMs with flow cytometry. Upon ZIKV^PR^ or ZIKV^U^ infection at 0.1 MOI, the mean percentage of dsRNA-positive MDMs was 7.37% (ZIKV^PR^-infected MDMs vs moDCs at 24hpi: *P*=0.0108) and 11.27% (ZIKV^U^-infected MDMs vs moDCs at 24hpi: *P*=0.0198) at 24hpi, which increased to 15.45% (ZIKV^PR^-infected MDMs vs moDCs at 48hpi: *P*=0.0089) and 15.88% (ZIKV^U^-infected MDMs vs moDCs at 48hpi: *P*=0.0312) at 48hpi, respectively (Figure 1C). Compared with viral dsRNA, the viral E protein was expressed at a lower percentage in ZIKV^PR^-infected and ZIKV^U^-infected MDMs at both 24hpi and 48hpi (Figure 1D). At 1.0 MOI, the expression of viral dsRNA and E protein in the infected MDMs and moDCs modestly increased in comparison to that infected at 0.1 MOI (Figure S1). Interestingly, the expression of viral dsRNA and E protein in ZIKV-infected MDMs were in general lower than those of ZIKV-infected moDCs (Figure 1 and Figure S1).

**Figure 2.**
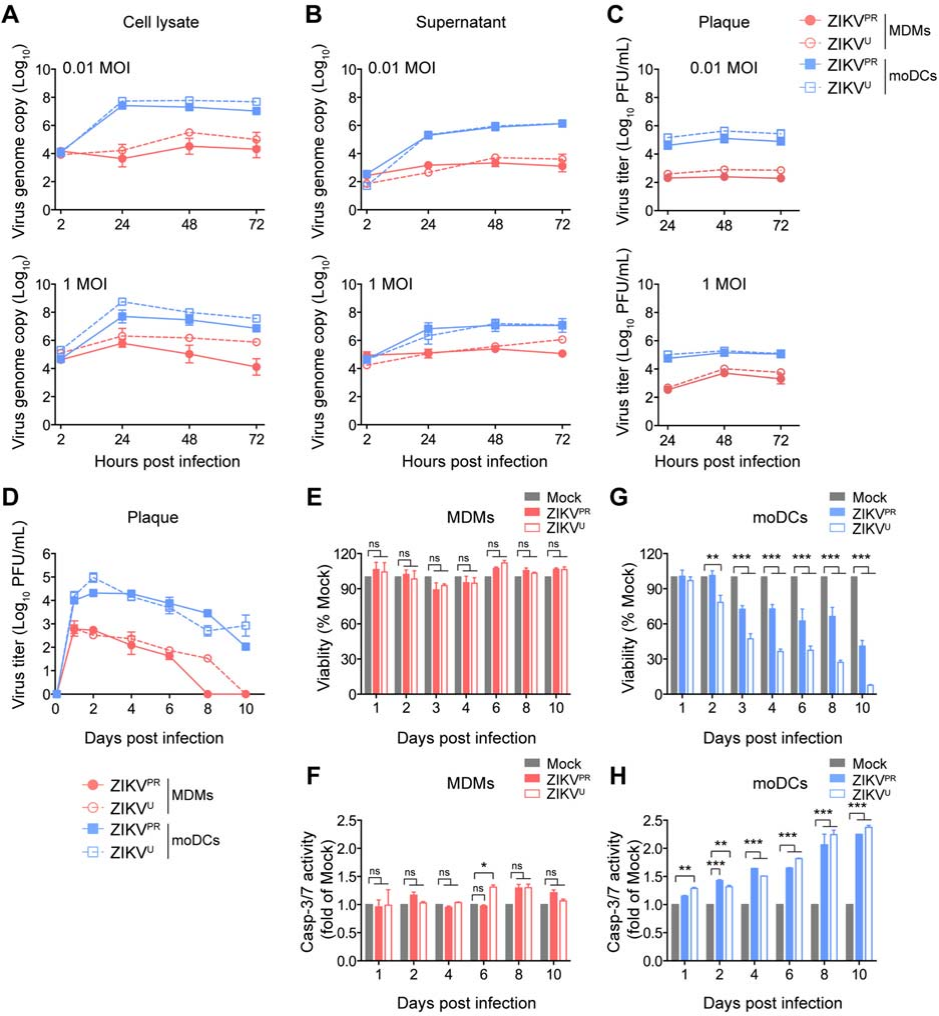
**MDMs but not moDCs restrict ZIKV replication.** MDMs and moDCs were infected with ZIKV^PR^ or ZIKV^U^ at an MOI of 0.01 or 1.0. Viral genome copy in the (**A**) cell lysates and (**B**) supernatants were determined by qRT-PCR. **(C)** The live infectious virus titer in the supernatants was measured by plaque assays. **(D)** MDMs and moDCs were infected with ZIKV^PR^ or ZIKV^U^ at an MOI of 0.1. The supernatants were harvested for determining the infectious virus titer by plaque assays at the indicated days post-infection. The (**E**) cell viability and (**F**) caspase-3/7 activities of uninfected and ZIKV-infected MDMs; and (**G**) cell viability and (**H**) caspase-3/7 activities of uninfected and ZIKV-infected moDCs were determined at the indicated time points by CellTiterGlo and CaspaseGlo 3/7 assays, respectively. Data represented mean and standard deviations from 3–6 donors. Statistical analyses in all panels were performed with two way-ANOVA and the differences were considered significant when *p* < 0.05. *p < 0.05,**p < 0.01, and ***p < 0.001.

Next, we examined the replication kinetics of ZIKV in MDMs and moDCs by determining the viral genome copy and infectious virus titer at different time points. In agreement with the flow cytometry results, moDCs supported robust ZIKV replication with a 4–5 log_10_ increase in viral genome copy (Figures 2A and 2B) over the 72 h incubation period. In contrast, ZIKV replicated modestly in MDMs with only 1–2 log_10_ increase in viral genome copy over the same period (Figures 2A and 2B). Similarly, the peak infectious virus titer reached approximately 10^5^–10^6^ PFU/mL in the infected moDCs, but only 10^2^–10^4^ PFU/mL in the infected MDMs, depending on the virus inoculum (Figure 2C). Notably, although the replication efficiency of ZIKV in moDCs was higher than that in MDMs, ZIKV replication in both cell types was less efficient than that in VeroE6 cells (Figure S2). In addition, the replication pattern of yellow fever virus (YFV) in MDMs, moDCs, and VeroE6 was largely comparable to that of ZIKV. Intriguingly, comparable levels of viral RNA were detected in the cell lysates of ZIKV-infected MDMs and moDCs at 2hpi, suggesting that the discrepancies in ZIKV replication were not due to virus entry (Figure 2A). Since the infectious virus titer remained detectable at 72hpi without significant reduction in both ZIKV-infected MDMs and moDCs, we further explored ZIKV infection in these cell types at later time points. As shown in Figure 2D, ZIKV-infected moDCs continued to release infectious virus particles that were largely sustained in the supernatants even at day 10 post infection, whereas the production of infectious virus particles was rapidly halted in ZIKV-infected MDMs and fell below the detection limit at day 8–10 post infection (Figure 2D). The decrease in virus titer in the infected MDMs was not due to ZIKV-induced cytotoxicity since the viability and caspase-3/7 activity of ZIKV-infected MDMs remained similar compared to the mock-infected cells (Figures 2E and 2F). Meanwhile, the viability of ZIKV-infected moDCs dropped to 40.93% and 7.57% of mock for ZIKV^PR^- and ZIKV^U^-infected moDCs at day 10 post infection, respectively (Figure 2G). Moreover, moDCs were susceptible to ZIKV-induced apoptosis as indicated by the significantly elevated caspase-3/7 activity (Figure 2H). Taken together, our data suggested that while moDCs supported ZIKV replication, ZIKV replication in MDMs was largely restricted.

### Restricted ZIKV replication in MDMs is not due to inefficient virus entry

**Figure 3.**
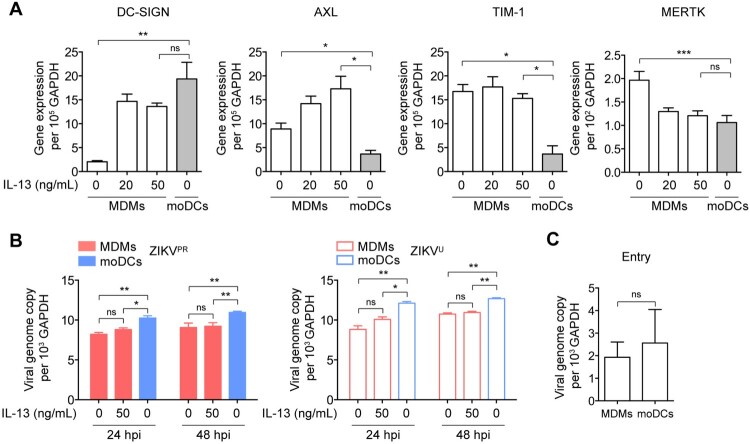
**Restricted ZIKV replication in MDMs is not due to inefficient virus entry.** (**A**) MDMs were untreated or treated with IL-13 (20 and 50 ng/mL) for 24 h and then subjected to determine the expression levels of ZIKV-related entry factors, including DC-SIGN, AXL, TIM-1, and MERTK by qRT-PCR. moDCs were included as a control. (**B**) MDMs were untreated or treated with IL-13 (50 ng/mL) for 24 h prior to infection with ZIKV^PR^ or ZIKV^U^ at an MOI of 0.01. The viral genome copy in the cell lysates was determined by qRT-PCR at 24hpi and 48hpi. (**C)** Viral entry capability in MDMs and moDCs were determined by qRT-PCR and normalized with GAPDH. Data represented mean and standard deviations from 3 donors. Statistical analyses in all panels were performed with one way-ANOVA and the differences were considered significant when *p* < 0.05. *p < 0.05, **p < 0.01, and ***p < 0.001. ns, not significant.

To further evaluate whether the restricted virus replication in MDMs is due to inefficient virus entry, we measured the endogenous expression levels of ZIKV-related entry factors in MDMs, including dendritic cell-specific intercellular adhesion molecule-3-grabbing non-integrin (DC-SIGN) [42], AXL receptor tyrosine kinase (AXL) [43-45], T-cell immunoglobulin and mucin domain 1 (TIM-1) [42], and MERTK [46]. Our data demonstrated that MDMs expressed significantly higher levels of AXL, TIM-1, and MERTK, but less DC-SIGN than moDCs (Figure 3A). We next tested whether the restricted ZIKV infection in MDMs was due to the lower expression level of DC-SIGN by treating MDMs with IL-13, which is known to upregulate DC-SIGN expression [47,48], followed by infecting the cells with ZIKV. As shown in Figure 3A, IL-13 treatment substantially upregulated the expression of DC-SIGN in MDMs to levels comparable with that in moDCs. Interestingly, despite a comparable expression level of DC-SIGN and higher expression levels of AXL and TIM-1, ZIKV replication remained significantly less efficient in MDMs than that in moDCs (Figure 3B). In addition, the difference in replication was not a result of differential entry efficiency as ZIKV entry in MDMs and moDCs at 1hpi was not significantly different (Figure 3C). Together, our data indicate that MDMs restrict ZIKV replication at post-entry events in the viral replication cycle.

### ZIKV infection does not antagonize type I IFN-mediated responses in MDMs

**Figure 4.**
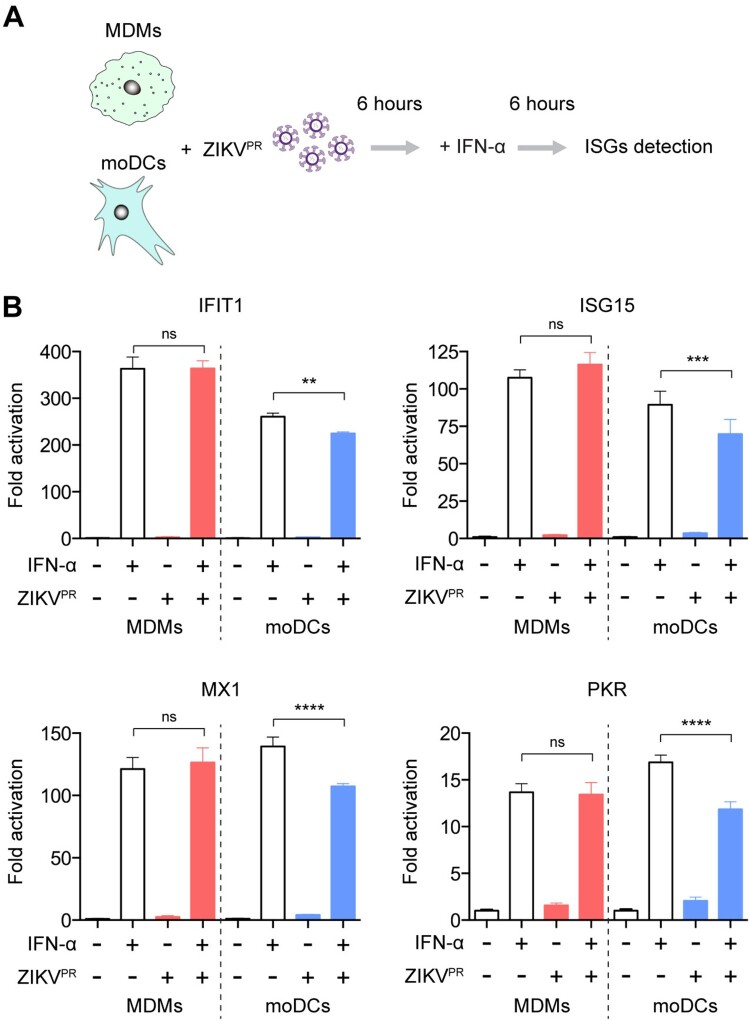
**ZIKV infection does not antagonize type I IFN-mediated responses in MDMs.** (**A**) Schematic illustration of human recombinant IFN-α treatment and ZIKV infection. (**B**) MDMs and moDCs were uninfected or infected with ZIKV^PR^ at an MOI of 10.0 for 6 h, followed by mock treatment or treatment with 1000U/mL of recombinant human IFN-α for 6 h. Cell lysates were collected for detection of ISGs induction using qRT-PCR, including IFIT1, ISG15, MX1, and PKR; the fold activations were calculated as compared to mock groups. Data represented mean and standard deviations from 3 donors. Statistical analyses in all panels were performed with one way-ANOVA and the differences were considered significant when *p* < 0.05. **p < 0.01, ***p < 0.001, and ****p < 0.0001. ns, not significant.

ZIKV has been demonstrated to antagonize the host IFN response to achieve optimal virus replication [22,23,26,49]. We next asked whether the restricted ZIKV replication could be attributed to inefficient viral antagonism of the IFN response in MDMs. MDMs or moDCs were infected with ZIKV^PR^ for 6 h at a multiplicity of infection (MOI) of 10.0 and subsequently treated with IFN-α for an additional 6 h (Figure 4A). The cell lysates were harvested to evaluate the expression of interferon-stimulated genes (ISGs), including IFIT1, ISG15, MX1, and PKR. IFN-α treatment upregulated the expressions of all four ISGs in MDMs and moDCs (Figure 4B). In ZIKV-infected moDCs, the upregulated expressions of ISGs through IFN-α treatment were significantly attenuated (IFIT1: from 260-fold to 224-fold; ISG15: from 89-fold to 69-fold; MX1: from 139-fold to 107-fold; PKR: from 17-fold to 12-fold). In stark contrast, ZIKV infection did not attenuate the expressions of any of the evaluated ISGs in MDMs (Figure 4B). These results suggest that ZIKV infection insufficiently antagonizes type I IFN-mediated antiviral responses in MDMs, which results in restricted viral replication in these cells.

### ZIKV infection does not inhibit the phosphorylation of STAT1 and STAT2 in MDMs

**Figure 5.**
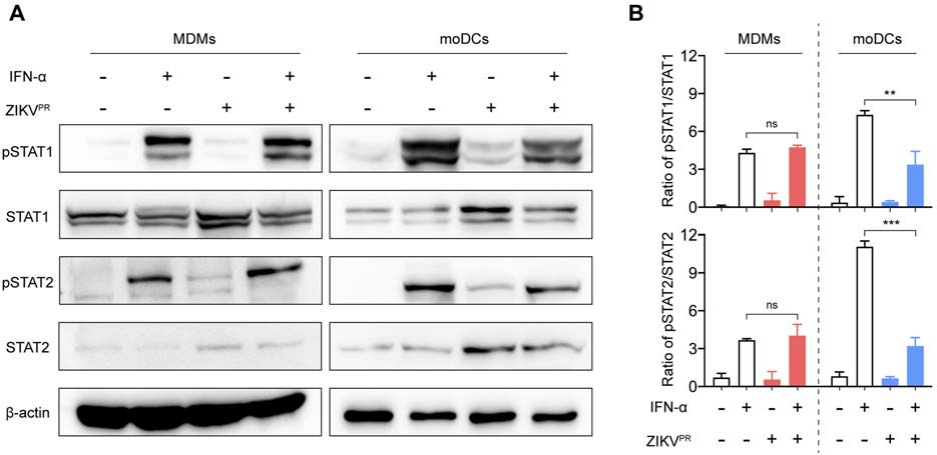
**ZIKV infection fails to inhibit the phosphorylation of STAT1 and STAT2 in MDMs. (A)** MDMs and moDCs were uninfected or infected with ZIKV^PR^ at an MOI of 1.0. At 48hpi, the cells were untreated or treated with 1000U/mL of recombinant human IFN-α for 40 min. The cell lysates were harvested for detecting expressions of pSTAT1, pSTAT2, STAT1, STAT2, and β-actin by Western blots. Representative blots represented data from three independent experiments. (**B**) Quantitation was calculated as the ratio of pSTAT/STAT protein from three independent experiments. Statistical analyses in all panels were performed with one way-ANOVA and the differences were considered significant when *p* < 0.05. **p < 0.01, and ***p < 0.001. ns, not significant.

It has been recently reported that ZIKV subverts type I IFN signaling through (i) virus-induced degradation of STAT2 [22-24] or (ii) blockade of STAT1 or STAT2 phosphorylation [24,26]. However, it remains unknown whether these IFN antagonism mechanisms are applicable to MDMs. To this end, we treated mock- or ZIKV^PR^-infected MDMs or moDCs with IFN-α for 30 min at 48hpi. The expression levels of STAT1, STAT2, and their phosphorylation were examined by Western blots. In mock-infected MDMs and moDCs, IFN-α treatment substantially promoted STAT1 and STAT2 phosphorylation. Upon ZIKV infection, the upregulated amount of pSTAT1 and pSTAT2 were significantly reduced in moDCs from 7.3 folds to 3.4 folds and 11.1 folds to 3.2 folds, respectively (Figure 5A and 5B). In contrast, ZIKV infection in MDMs did not counteract the phosphorylation of STAT1 or STAT2 stimulated by IFN-α treatment (Figure 5A and 5B). Previous studies reported that ZIKV NS5 played critical roles in viral antagonism of type I IFN signaling through degradation of STAT2. Thus, we examined the interaction of endogenous STAT2 with ZIKV NS5 in the infected MDMs and moDCs. As illustrated in Figure S3, we observed a much higher level of NS5 protein expression in the infected moDCs comparing to the infected MDMs. However, we did not detect any appreciable level of interaction between endogenous STAT2 and NS5 in both the infected MDMs and moDCs with the co-IP assays (Figure S3). Collectively, these results demonstrate that ZIKV does not efficiently antagonize the phosphorylation of STAT1 or STAT2 in MDMs, which may contribute to the attenuated type I IFN-mediated antiviral responses in these cells.

### Depletion of STAT2 but not STAT1 rescues ZIKV and YFV replication in MDMs

**Figure 6.**
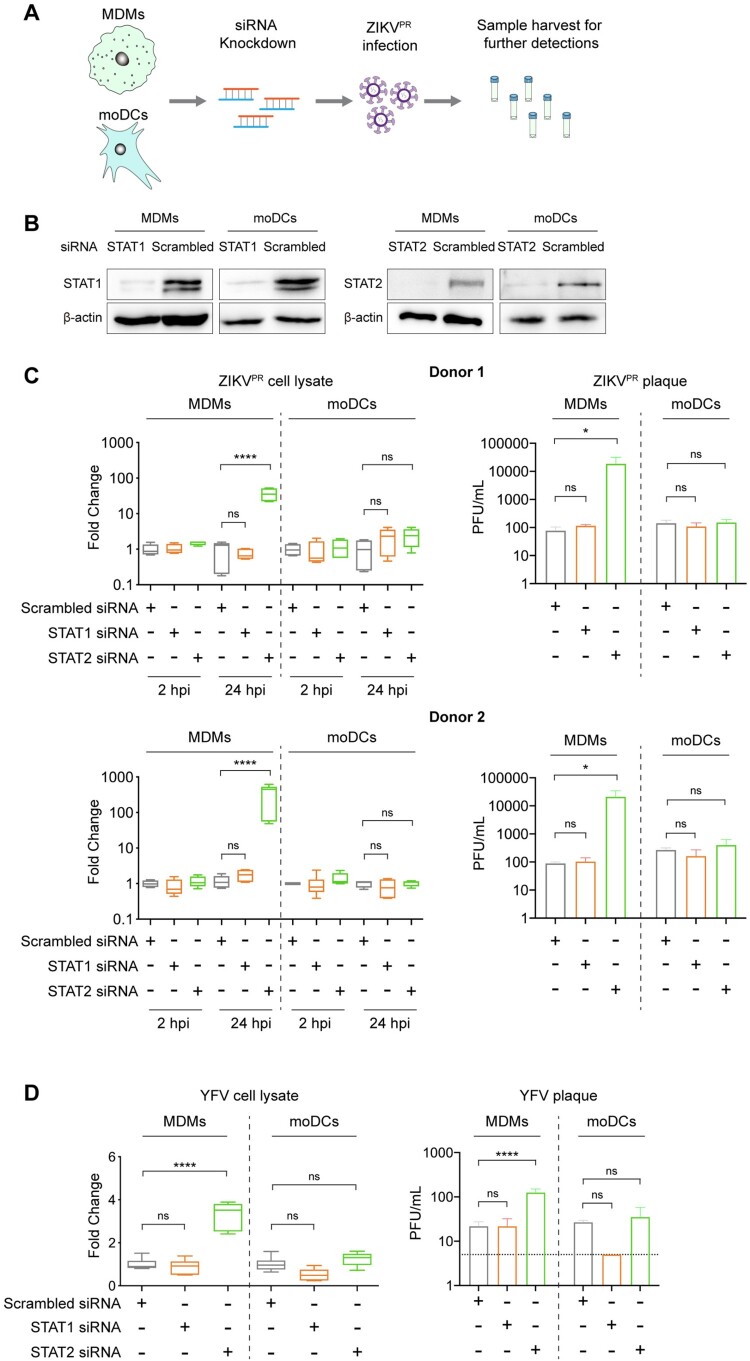
**Depletion of STAT2 but not STAT1 rescues ZIKV and YFV replication in MDMs.** (**A**) Schematic illustration of siRNA transfection and ZIKV infection. (**B**) MDMs and moDCs were transfected with scrambled siRNA or siRNA targeting STAT1 or STAT2. The knockdown efficiency of siRNA transfection was determined by Western blots. (**C**) MDMs and moDCs were generated from 2 donors. The cells were transfected with scrambled siRNA or siRNA targeting STAT1 or STAT2, followed by infection with ZIKV^PR^ at an MOI of 1.0. At 2hpi and 24hpi, the cell lysates and supernatants were harvested for detection of viral genome copy and live infectious virus titer by qRT-PCR and plaque assays, respectively. (**D**) MDMs and moDCs were transfected with the same procedure as mentioned above and then infected with YFV at an MOI of 1.0. The cell lysates and supernatants were collected for further detection at 24hpi. Statistical analyses in all panels were performed with one way-ANOVA and the differences were considered significant when *p* < 0.05. *p < 0.05, and ****p < 0.0001. ns, not significant.

Next, we performed loss-of-function studies to further investigate the role of STAT1 and STAT2 on ZIKV replication in MDMs. We transfected MDMs and moDCs with scrambled siRNA or siRNA targeting STAT1 or STAT2 (Figure 6A). At 24 h post-transfection, the siRNA-treated cells were infected with ZIKV^PR^. The cell lysates and supernatants were harvested for determining virus replication at 2hpi and 24hpi. Western blots showed that the knockdown efficiencies of STAT1 and STAT2 in MDMs were comparable to those in moDCs (Figure 6B). We further showed that the siRNA knockdown of STAT1 and STAT2 was specific, and was able to interfere with IFN signaling in MDMs and moDCs (Figure S4 and S5). Interestingly, the depletion of STAT1 did not modulate ZIKV replication in either MDMs or moDCs from both donors, indicating a dispensable role of STAT1 on regulating ZIKV replication in these two cell types (Figure 6C). Importantly, depletion of STAT2 in MDMs resulted in a 37-fold to 354-fold increase in viral genome copy and 2-log increase in infectious virus titer compared to scrambled siRNA-treated MDMs. In contrast, STAT2 knockdown in moDCs showed an insignificant impact on ZIKV replication when compared with the scrambled siRNA-treated moDCs (Figure 6C).

We further examined whether this observation could be applicable to other flaviviruses. In line with our results obtained using ZIKV, qRT-PCR and plaque assays demonstrated that the replication of YFV significantly increased in MDMs upon genetic depletion of STAT2 but not STAT1, whereas viral replication in moDCs was not significantly rescued after either STAT1 or STAT2 knockdown (Figure 6D). Overall, these results suggest that MDMs but not moDCs restrict ZIKV and YFV in a STAT2-dependent manner, implicating potential discrepancies of regulating viral replication in MDMs and moDCs.

## Discussion

Macrophages and dendritic cells are key sentinel cell types, playing critical roles in the host immune response against virus infection. However, the detailed interplay between human primary macrophages and ZIKV remains elusive. In this study, we demonstrated that ZIKV replication was largely restricted in MDMs but not in moDCs. The underlying mechanism of the restricted ZIKV replication in MDMs was not due to inadequate virus entry. Our data further illustrated that ZIKV infection in MDMs did not efficiently suppress type I IFN-mediated antiviral response and failed to antagonize STAT1 and STAT2 phosphorylation induced by IFN-α treatment. Moreover, depletion of STAT2 but not STAT1 substantially rescued ZIKV and YFV replication in MDMs. These findings suggest that MDMs restrict flavivirus replication in a STAT2-dependent manner that is not antagonized during virus infection. Overall, our study provided novel insights into the role of human macrophages in the restriction of flavivirus infection.

Macrophages and dendritic cells are reported to be susceptible to various flaviviruses. However, although mouse bone marrow-derived dendritic cells (bmDCs) and macrophages (bmMs) are permissive to JEV infection, the susceptibility of bmMs is much higher than that of bmDCs [12]. Interestingly, in comparison with human moDCs, human MDMs and mature dendritic cells (mDCs) are less susceptible to DENV infection [50]. Notably, the mechanism of the discrepant JEV and DENV replication between macrophages and dendritic cells was not explored in those studies. Here, we demonstrated that ZIKV replication was largely restricted in human primary MDMs but not in moDCs. In addition, we illustrated that the restricted ZIKV replication in MDMs occurred at post-entry events of the viral replication cycle. Since Ly6C^+^ blood monocytes enter the dermis and become MDMs or monocyte-derived tissue-resident macrophages, which are targeted by flaviviruses when the infected mosquitos bite [18,21], our findings suggested that MDMs may play indispensable roles in restricting the initial wave of ZIKV infection to limit viral dissemination in the infected host.

Flaviviruses have evolved diverse IFN antagonism mechanisms targeting multiple stages of the IFN pathway to facilitate virus replication and spread. The NS5 of many flaviviruses, such as DENV and YFV, interacts and promotes STAT2 degradation through UBR4 or binding to other E3 ligase TRIM23, thereby suppressing the antiviral responses [51,52]. Recent studies reported that ZIKV infection antagonizes type I IFN signaling by targeting STAT2 for proteasomal degradation [22,23]. At the same time, viral antagonism of type I IFN has been demonstrated to be cell type-dependent, which could determine the susceptibility of viral infection and in turn affect tissue tropism [53,54]. In the present study, ZIKV infection was shown to suppress type I IFN-mediated response and inhibited phosphorylation of STAT1 and STAT2 in moDCs, which is in line with previous findings [26]. However, our data illustrated that the ZIKV-mediated inhibitory effect on IFN response was surprisingly not observed in MDMs, which contributed to inefficient virus replication in these cells. Previous studies reported that ZIKV NS5 played critical roles in antagonizing type I IFN signaling through degradation of STAT2. However, NS5 expression in the infected MDMs was inefficient and we did not detect any interaction between endogenous STAT2 and NS5 in the infected MDMs. The low level of NS5 in the infected MDMs may be due to the lack of virus replication in these cells, which should be recognized as a potential problem in interpreting the MDM results. Alternatively, the restricted viral replication may have resulted in the low expression level of NS5 in ZIKV-infected MDMs. Further studies should be conducted to investigate the detailed mechanisms of the cell type-dependent IFN-antagonism against ZIKV infection.

STAT1 and STAT2 are critical transcription factors required for the transcriptional activation of IFN-stimulated antiviral genes in type I, II, and III IFN signaling [55]. However, STAT2 is relatively diverse across different species [56,57]. In addition, although STAT1 and STAT2 interact with each other and lead to the activation of downstream antiviral response, STAT2 is likely a key mediator in the host defense mechanism. STAT2-mediated antiviral responses attenuate DENV infection in mice even in the absence of STAT1 [58]. Further, STAT2 can overcome mouse cytomegalovirus (MCMV) pM27-mediated degradation to limit the virus spread in mice with STAT2-dependent immune responses [59]. Here, we provided the first evidence that demonstrated the essential role of STAT2 against ZIKV and YFV infection in MDMs. The exact mechanism of how STAT2 overcomes ZIKV antagonism in MDMs should be further explored.

Overall, our findings provided novel insights into how ZIKV differentially interacts with the two sentinel cell types in the host immune system and underscoring the restriction role of MDMs against ZIKV infection.

## Supplementary Material

TEMI-2020-1858_Supplementary_Figure_legends.docx

TEMI_2020_1858_Figure_S5.tif

TEMI_2020_1858_Figure_S4.tif

TEMI_2020_1858_Figure_S3.tif

TEMI_2020_1858_Figure_S2.tif

TEMI_2020_1858_Figure_S1.tif
