# Supplementary material for: STAT2-dependent restriction of Zika virus by human macrophages but not dendritic cells
Source: Emerg Microbes Infect. 2021 Jun 8;10(1):1024–37. doi: 10.1080/22221751.2021.1929503 (PMC8205058; doi:10.1080/22221751.2021.1929503)
Supplement: TEMI-2020-1858_Supplementary_Figure_legends.docx [file TEMI_A_1929503_SM9971.docx]

**Supplementary figure legends**

**Figure S1. MDMs are susceptible to ZIKV infection.** Human monocyte-derived macrophages (MDMs) and dendritic cells (moDCs) were uninfected or infected with ZIKV^PR^ or ZIKV^U^ at an MOI of 1.00. At 24hpi and 48hpi, ZIKV-infected and mock-infected cells were fixed and stained with J2 or 4G2, and subsequently applied to flow cytometry to determine expression levels of viral dsRNA and E protein. The dot-plot showed data of one representative donor. The histogram showed the mean values and standard deviations from three donors. Statistical analysis in all panels were performed with Student t test and the differences were considered significant when p<0.05. *p<0.05, **p<0.01. ns, not significant.

**Figure S2. MDMs but not moDCs restrict ZIKV replication.** MDMs, moDCs and VeroE6 were infected with ZIKV^PR^, ZIKV^U^, or YFV at an MOI of 1.00. The supernatants were collected in the indicated time points for determining the live infectious virus titer by plaque assays.

**Figure S3. ZIKV NS5 does not interact with endogenous STAT2 of MDMs and moDCs.** MDMs and moDCs were uninfected or infected with ZIKV^PR^ at an MOI of 5. After 24hpi, the cell lysates were immunoprecipitated with either anti-ZIKV NS5 antibody or anti-STAT2 antibody pre-crosslinked onto Protein A/G Magnetic beads. The precipitated protein complex was detected using the anti-STAT2 antibody or the anti-ZIKV NS5 antibody, respectively. The expression of ZIKV NS5, STAT2, and β-actin were detected by Western blots. WCE: whole cell lysates.

**Figure S4. Transfection with siRNA does not induce off-target effects.** MDMs and moDCs were transfected with scrambled siRNA or siRNA targeting STAT1 or STAT2. The cell lysates were collected to determine the knockdown efficiency of siRNA transfection by Western blots.

**Figure S5. The depletion of STAT2 but not STAT1 reduces induction of ISGs in MDMs and moDCs.** MDMs and moDCs were transfected with scrambled siRNA or siRNA targeting STAT1 or STAT2, and followed by transfection with Poly IC or with control. After 12h, the cell lysates were harvested for detecting host gene expressions by qRT-PCR, including IFIT1, MX1, ISG15, IRF1, and IP10. Data represented mean and standard deviation from two donors. Statistical significance was determined with two way-ANOVA and the differences were considered significant when p<0.05. **p<0.01, ***p<0.001, and ****p < 0.0001. ns, not significant. ud, undetectable.
