# Supplementary figures and images for: STAT2-dependent restriction of Zika virus by human macrophages but not dendritic cells
Source: Emerg Microbes Infect. 2021 Jun 8;10(1):1024–37. doi: 10.1080/22221751.2021.1929503 (PMC8205058; doi:10.1080/22221751.2021.1929503)

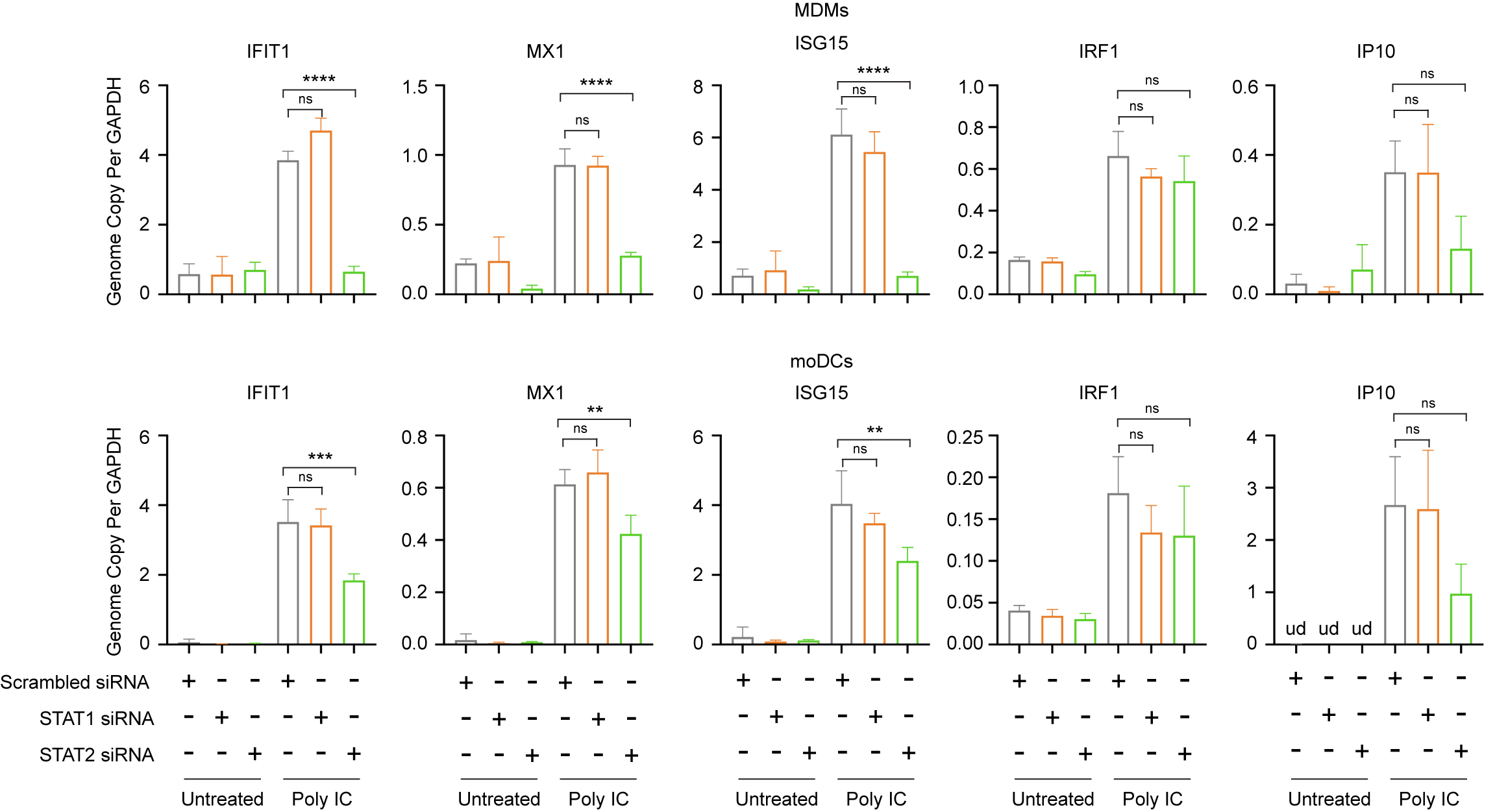

Supplement: TEMI_2020_1858_Figure_S5.tif [file TEMI_A_1929503_SM9970.tif]

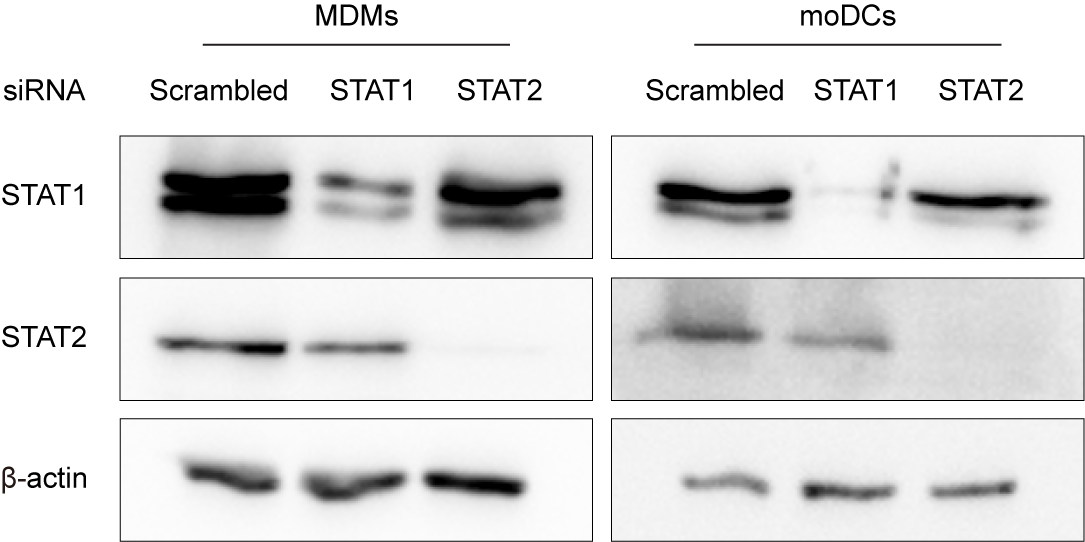

Supplement: TEMI_2020_1858_Figure_S4.tif [file TEMI_A_1929503_SM9969.tif]

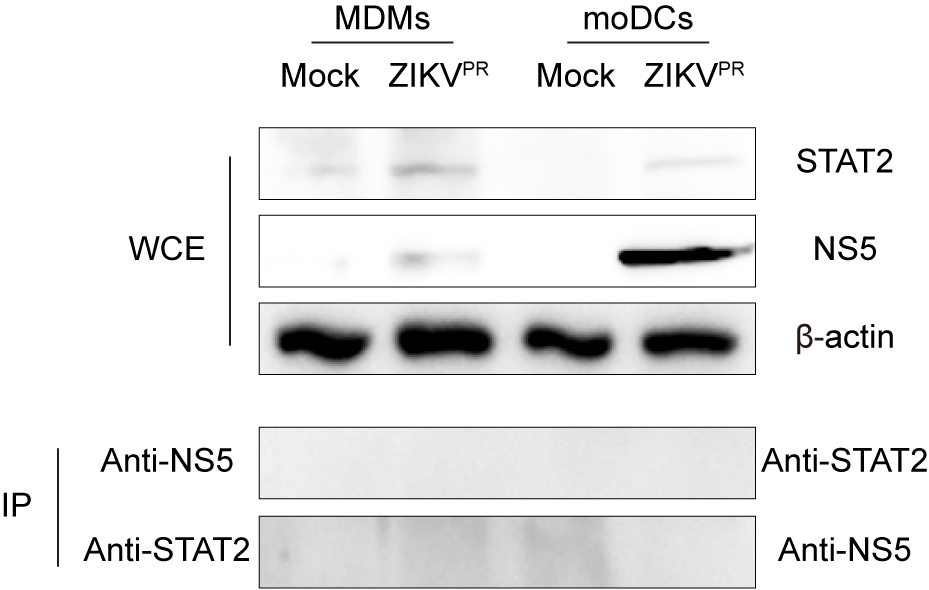

Supplement: TEMI_2020_1858_Figure_S3.tif [file TEMI_A_1929503_SM9968.tif]

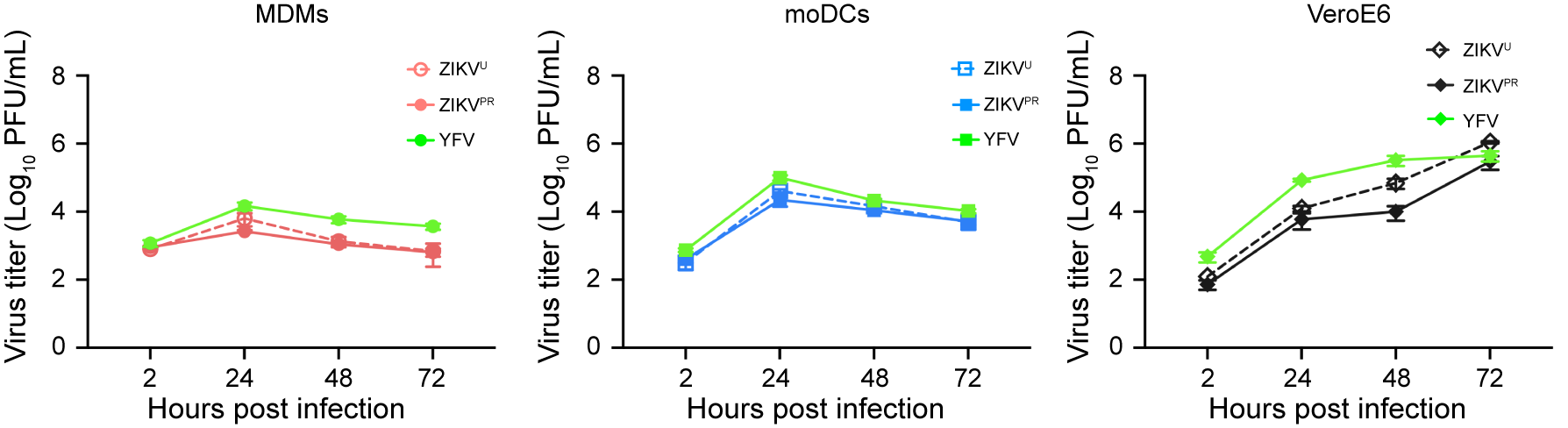

Supplement: TEMI_2020_1858_Figure_S2.tif [file TEMI_A_1929503_SM9967.tif]

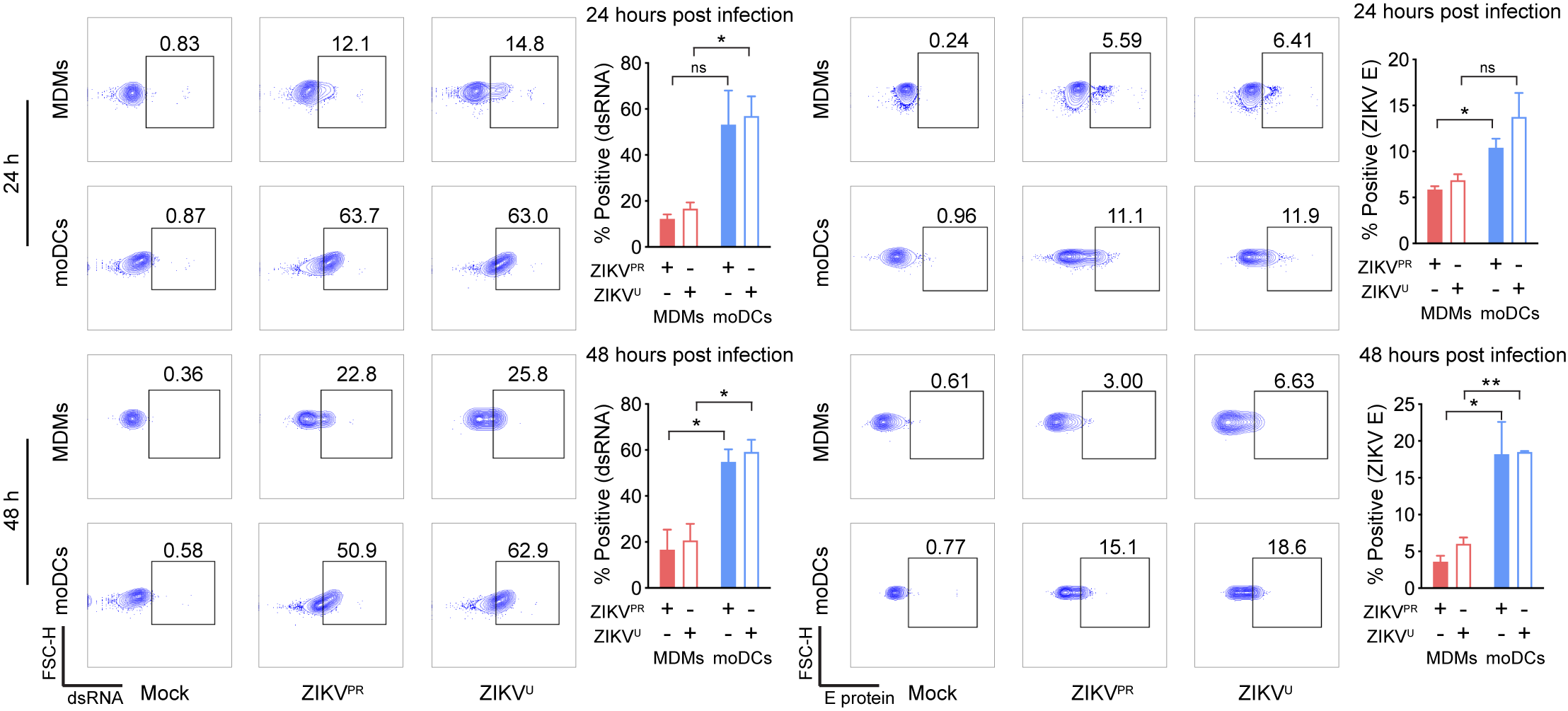

Supplement: TEMI_2020_1858_Figure_S1.tif [file TEMI_A_1929503_SM9966.tif]
